# Association between environmental stress factors, salivary cortisol level and dental caries in Egyptian preschool children: a case-control study

**DOI:** 10.1038/s41598-025-94327-0

**Published:** 2025-04-01

**Authors:** Yosra Ahmed Hussein, Rania Hussein Refai, Mai M. K. Hussein, Mamdouh Hanafy Abdou, Magdy Mohamed El Bordini, Ola Mohamed Ewais, Mohamed Fakhry Hussein

**Affiliations:** 1 Dental Unit, Medical Research Institute, Alexandria University, Alexandria, Egypt; 2 Department of Medicine Supply and Pharmacy, Alexandria University Hospitals, Alexandria University, Alexandria, Egypt; 3 Clinical Research Administration, Alexandria Directorate of Health Affairs, Alexandria, Egypt; 4 Ministry of Health and Population, Cairo, Egypt; 5 Department of Occupational Health and Industrial Medicine, High Institute of Public Health, Alexandria University, Alexandria, Egypt; 6 Department of Clinical Pathology, Faculty of Medicine, Alexandria University, Alexandria, Egypt

**Keywords:** Preschool children, Early childhood caries, Environmental risk factors, Salivary cortisol, Stress, Biomarkers, Health care, Medical research, Risk factors

## Abstract

Dental caries remains the most common chronic disease in childhood. This study aimed to identify environmental stressors in children and estimate the association between salivary cortisol level as a stress biomarker and childhood caries. We conducted a case-control study involving 80 Egyptian children. The cases were 40 children with early childhood caries (ECC). The controls included 40 caries-free children. Data were collected from the parents or caregivers of the children using a questionnaire. The clinical examination included caries experience and an oral hygiene index. Saliva samples were collected and tested for unbounded cortisol levels. The results from the current study showed that the mean age for participants was 5.09 ± 0.66 years. Twenty-one of the children with ECC were girls. In multivariate regression, there is a significant association between ECC and elevated salivary cortisol levels (> 0.57 µg/dl) with an adjusted odds ratio (AOR) of 9.649 (*p* = 0.003), father smoking (AOR = 5.791, *p* = 0.030), sleeping with a bottle or food in the mouth (AOR = 8.481, *p* = 0.027), and poor oral hygiene evaluated through the Silness-Löe Plaque Index (AOR = 6.681, *p* = 0.001). As a conclusion, the study indicates that salivary cortisol is an independent stress biomarker in relation to early childhood caries. Salivary cortisol as a stress biomarker can help clinicians provide an additional assessment tool for identifying caries risk in children.

**Supplementary Information:**

The online version contains supplementary material available at 10.1038/s41598-025-94327-0.

## Introduction

Dental caries remains the most common chronic disease in childhood. It is five times more common than asthma and seven times more common than hay fever^1^. It is considered a multifactorial complex disease that is influenced by a variety of factors, including diet, oral hygiene, socioeconomic status, genetic predisposition, and environmental factors. The interaction of these factors contributes to its development and progression^2,3^. Global estimates of dental caries prevalence in primary and permanent teeth in 2020 were 46.2% and 53.8%, respectively^4^. The prevalence in developing countries was estimated to be as high as 50–80%^5^. Untreated carious lesions can lead to expensive treatment, disruption of growth and development, pain, and life-threatening infections^6^.

Early childhood caries (ECC) has been defined as the presence of one or more decayed (non-cavitated or cavitated), missing (due to caries), or filled tooth surfaces in any primary tooth in a child 71 months of age or younger^7^. Early childhood caries is linked to several factors, including infant feeding practices such as prolonged bottle-feeding, especially with sugary liquids; early bacterial exposures, such as Mutans Streptococci (MS) often transmitted by caregivers; genetic predisposition that influences susceptibility to caries; environmental risk factors; and socioeconomic conditions, where low socioeconomic status can limit access to dental care and healthy foods, thus increasing the risk of caries. Deciduous teeth are more susceptible to caries than permanent teeth due to several factors including their thinner enamel and dentin that make them more vulnerable to acid attacks, the less organized structure of their enamel that weakens their resistance to decay. additionally, their higher carbonate content that can compromise their structural integrity. Finally, their shorter development period that can lead to less mineralization and increased caries risk^8,9^. The direct consequences of ECC are pain and infection. These can affect the child’s ability to eat and subsequently reduce growth and weight gain due to insufficient food consumption to meet the metabolic and growth needs of children. Furthermore, its consequences can affect the immediate and long-term quality of life of the child and family^10,11^.

Previous studies have reported that stress is a significant factor influencing overall health, and has been linked to increased dental caries risk. Increased cortisol is an important biomarker of stress response and has been associated with a higher susceptibility to dental caries^12^. Cortisol can induce atrophy of the salivary glands, reducing saliva production (quantity) and altering its composition (quality). These salivary changes may result in increased formation and adherence of cariogenic biofilm, potentially having a detrimental effect on teeth^13^. Furthermore, previous studies have reported that stress hormones such as cortisol can suppress the immune response, inhibiting the activity of essential antimicrobial agents in saliva such as salivary Immunoglobulin A, lactoferrin, lysozyme, and lactoperoxidase, further weakening oral defenses^14^.

Additionally, environmental and psychosocial stressors, such as poverty, single-parent households, low parental education levels, parental smoking, and academic challenges, can significantly elevate cortisol levels in children. Such children may eventually develop anxiety, making them prone to dental decay and other medical conditions^15^. Agarwal et al. (2023) reported greater prevalence of proximal caries in children with higher anxiety and stress levels in comparison to their counterparts^16^. Additionally, unhealthy coping mechanisms, like excessive mobile phone use, can exacerbate oxidative stress, potentially augmenting caries risk^17^. This complex interplay of biological, environmental, and psychosocial factors underscores the importance of addressing stress, which plays a substantial role in caries risk in children.

According to a WHO study, the prevalence of dental caries in Egypt is estimated to be 70%^18^. A 2019 Egyptian cross-sectional study reported a higher prevalence of dental caries in primary teeth compared to permanent teeth, with 74% of children affected^19^. Because of the scarcity of literature on the relationship between environmental factors and salivary cortisol levels in children with dental caries in Egypt, the current study is designed to assess this relationship. This study aimed to identify environmental stressors in a child’s everyday life that contribute to dental caries in children and to measure the salivary cortisol level as a stress biomarker for childhood caries.

## Methods

### Study design and setting

To evaluate the environmental stressors for dental caries in children and to measure the salivary cortisol level as a stress biomarker for childhood caries, a case-control study was conducted among children aged 4 to less than 6 years. The study was conducted at preschool nurseries in Alexandria and at the dental clinic of the Medical Research Institute, Alexandria University, from July 2022 to May 2023.

### Sample size and design

Based on the case-control study of Rai et al. (2010), the mean difference between cortisol levels in children with caries and caries-free children was 0.69 μg/dl, and the SD of children with caries was 1.344 μg/dl, SD of caries-free children was 0.337 μg/dl. Using Alpha error = 5% and beta error = 20%, the minimum required sample size was estimated to be 66 children, which was increased to 80, 40 for each group^20^.

The sample size was calculated using G. Power software. The cluster sample technique was used to select two districts out of ten administrative districts in Alexandria Governorate. From each district, 5 nurseries were chosen randomly, and 4 cases and 4 controls were selected randomly from each nursery.

### Study population

Forty children with caries and an equal number of caries-free children matched for age and gender were included. Cases were identified by a pediatric dentist based on clear diagnostic criteria for dental caries. Matched controls with no history of/or current diagnosis of dental caries were then selected from the same pool of population. Cases and controls were selected according to the following criteria:

Inclusion criteria included (1) Child aged 4 to < 6 years; (2) Only one child was chosen from each family.

Exclusion criteria included (1) Children with systemic diseases or special health care needs; (2) Children taking medications that interfere with measures of salivary cortisol.

The data collection tools:

#### Interview questionnaire

Data on past exposures were collected from the parents or caregivers of the children enrolled in the present study using a pre-structured, pre-coded interview questionnaire. The items of the questionnaire were acquired after meticulous reviewing of the literature and were obtained from previously validated research questionnaires^21–23^. Additionally, it was assessed by experts in dentistry and public health to be sure of the content validity. The English questionnaire was translated both forward and backward by native speakers who are specialists in public health.

The questionnaire included socio-demographic data comprising parental occupation and education. It also involved potential socio-environmental stressors in the child’s everyday life, including socio-economic situation, marital conflict or violence, child abuse or negligence, the death of a family member, and parental smoking. The kindergarten and school environment included being bullied by other children at school, peer pressure, losing games, having difficulties making friends or racism, having learning disabilities, moving to another school, bad grades, and too much homework. Dietary habits and dental hygiene were included in the questionnaire (Supplementary file I). The sleeping pattern of the child was obtained through the Epworth Sleepiness Scale for Children and Adolescents (ESS-CHAD)^24^ (Supplementary file II). Medical and dental histories were searched for any significant findings.

#### Clinical examination

##### Caries experience

The examination was performed using a plane mirror and dental explorer under daylight without drying to assess caries according to WHO criteria. The condition of each surface was recorded as sound, decayed, filled, or missing using the decayed, missing, and filled teeth (dmft) index. The individual dmft value is the sum of the number of decayed, missed due to caries, and filled teeth^25^. Those without caries (zero score) were considered control participants, and others were recruited in the case group.

##### Oral hygiene (Silness-Löe plaque Index)

The Silness-Löe Plaque Index was used to assess the thickness of plaque at the gingival area of index teeth. The measurement of the state of oral hygiene by the Silness-Löe Plaque Index is based on recording both soft debris and mineralized deposits on the index teeth. The scores from the four areas of the tooth are added and divided by four in order to give the plaque index for tooth^26^ (Supplementary file III).

#### Assessment of unbound cortisol level in saliva

##### Saliva collection

To standardize the time of collection, the saliva samples were always collected from children at the same appointment hours. Saliva sampling for each child was scheduled at morning appointments, between 9 a.m. and 12 p.m^27^.

##### Specimen Preparation

Eating, drinking, chewing gum, or brushing teeth were avoided for 30 min before saliva sampling. It was recommended to rinse the mouth thoroughly with cold water for 5 min before sampling. Sample collection was postponed if oral diseases, inflammation, or lesions existed that would be a source of blood contamination. If there was visible blood contamination in the patient’s saliva specimen, it was discarded. The sampling device was rinsed with water, waited for 10 min, and a new sample was taken.

The collected samples were transferred within a maximum of an hour to the laboratory of the Clinical Pathology Department, Faculty of Medicine, Alexandria University, to be frozen at -20^o^C and stored until processing time. The average absorbance values for each set of standards, controls, and patient samples were calculated. A calibration curve was constructed, and the results were interpolated from the curve^28^.

### Ethical approval

The study has obtained the approval of the Ethics Committee of the High Institute of Public Health, Alexandria University, Egypt, for conducting the research. The researchers complied with the International Guidelines for Research Ethics. A written informed consent was taken from the caregivers of the children after an explanation of the purpose and benefits of the research. Anonymity and confidentiality were ensured. All methods were performed in accordance with the relevant guidelines and regulations.

### Statistical analysis

According to the present research question, lifestyle factors and cortisol level were the exposures, while caries and plaque index were the outcomes. Descriptive statistics were calculated as frequencies and percentages for qualitative variables and means and standard deviations for quantitative variables. Children with ECC and caries-free children were compared regarding sociodemographic factors, oral health practices, dietary habits, and plaque index using the t-test, chi-square, Fisher exact, and Mann-Whitney U tests. Univariate regression analysis investigated the association between environmental stressors, free salivary cortisol (exposures), and the presence of ECC (outcome). Variables with significant associations were entered into a multivariate logistic regression analysis. Odds ratios (OR) and 95% confidence intervals (CI) were calculated to determine the significant risk factors for ECC. The significance level was set at 5%. SPSS version 17.0 was used for statistical analysis.

## Results

### Socio-demographic characteristics of participants

Table 1 represents the socio-demographic characteristics of both groups. The median age was 5 years old. No statistically significant differences existed between the two groups (children with ECC and caries-free children) as regards age or gender (*p* = 0.613 and 0.655 respectively), indicating homogeneity between the two groups. 55% of the children with ECC were from the East and Middle districts of Alexandria, while most of the caries-free children (67.5%) were from the El Montazah and East districts. No statistically significant differences existed between the two groups as regards the address of the children (*p* = 0.627).

**Table 1:** Distribution between the two studied groups according to their socio-demographic characteristics (Medical Research Institute, 2023).

Socio-demographic characteristics	Cases (*n* = 40)		Controls (*n* = 40)		Test of significance	*p*-value
	*N*	%	*N*	%		
Age (years) mean ± SD	5.09 ± 0.66		5.01 ± 0.66		t = 0.507	0.613
Sex						
Male	19	47.5	21	52.5	**χ**^**2**^ = 0.2	0.655
Female	21	52.5	19	47.5		
Residence						
El Montazah district	8	20.0	10	25.0	**χ**^**2**^ = 5.669	^MC^p= 0.627
East district	12	30.0	17	42.5		
Middle district	10	25.0	8	20.0		
Other districts	10	25.0	5	12.5		
Order of birth among siblings						
First	10	25.0	14	35.0	**χ**^**2**^ = 3.995	^MC^p= 0.273
Second	16	40.0	14	35.0		
Third	7	17.5	10	25.0		
More	7	17.5	2	5.0		
Maternal education						
Illiterate + Read & write	5	12.5	3	7.5	**χ**^**2**^ = 4.503	^MC^p= 0.213
Primary + middle + high	14	35.0	11	27.5		
Above average qualification	11	27.5	7	17.5		
University + Postgraduate	10	25.0	19	47.5		
Mother working						
Non-working	19	47.5	14	35.0	**χ**^**2**^ = 6.684^*^	0.035^*^
Clerical	5	12.5	15	37.5		
Heavy working	16	40.0	11	27.5		
Paternal education						
Illiterate + Read & write	6	15.0	4	10.0	**χ**^**2**^ = 8.480^*^	0.037^*^
Primary + middle + high	10	25.0	10	25.0		
Above average qualification	17	42.5	8	20.0		
University + Postgraduate	7	17.5	18	45.0		
Father working						
Non-working	4	10.0	2	5.0	**χ**^**2**^ = 0.761	^MC^p= 0.823
Clerical	12	30.0	12	30.0		
Heavy working	24	60.0	26	65.0		

χ^2^: Chi square test, MC: Monte Carlo, t: t test, p: p value for comparing between the studied groups, *Statistically significant at *p* ≤ 0.05.

There was no statistically significant difference as regards the order of birth of the child among siblings in both groups (*p* = 0.273). However, there was a statistically significant difference between the two groups as regards the mother’s occupation (*p* = 0.035). Most of the mothers of children with ECC were either non-working (47.5%) or had heavy work (40%). On the other hand, 37.5% of the mothers of caries-free children have clerical work. Nearly half of the control group (45%) had highly educated fathers (university or postgraduate education) compared to only 17.5% of the case group, and this difference was statistically significant (*p* = 0.037).

### Potential socio-environmental stressors in the child’s everyday life

Table 2 demonstrates the comparison between the two studied groups according to potential socio-environmental stressors in the child’s everyday life (as stated by the mother or caregiver). The family income was not enough for 37.5% of the families of children with ECC and 25% of the families of caries-free children. However, the difference was not significant (*p* = 0.505).

**Table 2:** Comparison between the two studied groups according to potential socio-environmental stressors in the child’s everyday life (Medical Research Institute, 2023).

	Cases (*n* = 40)		Controls (*n* = 40)		Test of significance	*p*-value
	*N*	%	*N*	%		
Potential socio-environmental stressors						
Family income						
Enough and exceed	4	10.0	4	10.0	χ^2^= 1.58	^MC^p= 0.505
Enough	21	52.5	26	65.0		
Not enough	15	37.5	10	25.0		
Parental depression	12	30.0	10	25.0	χ^2^ = 0.25	0.617
Parental separation/ conflict/Divorce	4	10.0	3	7.5	χ^2^ = 0.16	^FE^p= 1.000
Parental violence	7	17.5	3	7.5	χ^2^= 1.83	0.176
Child abuse or negligence	2	5.0	1	2.5	χ^2^ = 0.346	^FE^p= 1.000
New sibling to the child	13	32.5	14	35.0	χ^2^ = 0.06	0.813
Father smoking	23	57.5	14	35.0	χ^2^ = 4.07	0.044*
Kindergarten and school environment						
Being bullied by other children at school	10	25.0	6	15.0	χ^2^ = 1.25	0.264
Having difficulties making friends or racism	8	20.0	6	15.0	χ^2^ = 0.35	0.556
Having learning disabilities	13	32.5	5	12.5	χ^2^ = 4.59	0.032*
Moving to another school	6	15.0	3	7.5	χ^2^= 1.13	^FE^p= 0.481
Bad grades	4	10.0	3	7.5	χ^2^= 0.16	^FE^p= 1.000
Too much homework	11	27.5	8	20.0	χ^2^= 0.62	0.431
Modified pediatric Epworth sleepiness scale						
Total Epworth score						
Normal (0–10)	22	55.0	34	85.0	χ^2^ = 9.77	^MC^p= 0.012^*^
Mild (11–14)	12	30.0	3	7.5		
Moderate (15–17)	4	10.0	3	7.5		
Severe (18–24)	2	5.0	0	0.0		
Min. – Max.	0.0–18.0		0.0–16.0		U = 518.50	0.007^*^
Mean ± SD.	9.10 ± 5.15		6.08 ± 4.47			
Median (IQR)	10.0 (4.50 − 12.50)		6.0 (2.0–9.0)			

χ^2^: Chi square test, MC: Monte Carlo, FE: Fisher Exact, U: Mann Whitney test, p: p value for comparing between the studied groups, *: Statistically significant at *p* ≤ 0.05.

There was no significant difference concerning the incidence of parental depression (30% in cases vs. 25% in controls) (*p* = 0.617) or parental separation (10% in cases vs. 7.5% in controls) (*p* = 1.00). Although parental violence was reported by 17.5% of children with ECC compared to 7.5% of caries free children, the difference did not reach a statistically significant level (*p* = 0.176).

Having learning disabilities was reported by 32.5% of children with ECC, compared to 12.5% of caries-free children. There is a statistically significant difference between the two groups as regards having learning disabilities (*p* = 0.032).

On the other hand, there was no statistically significant difference between the two groups in relation to other factors, including being bullied by other children at school (25% in cases, 15% in controls, *p* = 0.264), having difficulties making friends or racism (20% in cases, 15% in control, *p* = 0.556), moving to another school (15% in cases, 7.5% in controls, *p* = 0.481), bad grades (10% in cases, 7.5% in controls, *p* = 1.000) and too much homework (27% in cases, 20% in controls, *p* = 0.431). Most fathers of children with ECC (57.5%) were smokers, while only 35% of the fathers of caries-free children were smokers. The difference was statistically significant (*p* = 0.044).

Table 2 also shows the comparison between the two groups in relation to the Epworth Sleepiness Scale for Children and Adolescents (ESS-CHAD). There were mild sleeping problems in 30% of children with ECC and in only 7.5% of caries-free children (*p* = 0.012), while moderate sleepiness was detected in 10% of children with ECC and in only 7.5% of caries-free children. The mean ESS-CHAD in children with ECC was significantly higher (9.10 ± 5.15) than that of caries-free children (6.08 ± 4.47) (*p* = 0.007).

A comparison between the two groups in relation to dental history and dietary habits is shown in Table 3. Exclusive breast feeding was reported by 72.5% of children with ECC, compared to 75% of caries-free children. The difference was not statistically significant (*p* = 0.393). Sleeping with a bottle or food in the mouth was reported by 25% of children with ECC compared to only 7.5% of caries-free children; the difference was statistically significant (*p* = 0.034).

**Table 3:** Comparison between the two studied groups according to dental history and dietary habits (Medical Research Institute, 2023).

Dental history & dietary habits	Cases (*n* = 40)		Control (*n* = 40)		χ^2^	*p*-value
	*N*	%	*N*	%		
Previous dental visits						
No	19	47.5	27	67.5	3.274	0.070
Yes	21	52.5	13	32.5		
Infant milk feeding habit						
Breast feeding	29	72.5	30	75.0	1.937	^MC^p= 0.393
Bottle feeding	7	17.5	9	22.5		
Mixed	4	10.0	1	2.5		
Sleeping with bottle /food in the mouth						
No	30	75.0	37	92.5	4.501^*^	0.034^*^
Yes	10	25.0	3	7.5		
Wake up to eat /drink juice						
No	13	32.5	19	47.5	1.875	0.171
Yes	27	67.5	21	52.5		
Cleaning teeth using toothbrush						
No	7	17.5	7	17.5	0.096	0.953
Once daily	26	65.0	27	67.5		
Twice daily	7	17.5	6	15.0		
Brushing teeth before bedtime	(*n* = 33)		(*n* = 33)			
No	14	42.4	8	24.2	2.455	0.117
Yes	19	57.6	25	75.8		
Using fluoride toothpaste						
No	15	37.5	17	42.5	0.208	0.648
Yes	25	62.5	23	57.5		
Parental supervision on teeth brushing						
No	13	32.5	15	37.5	0.220	0.639
Yes	27	67.5	25	62.5		

χ^2^: Chi square test, MC: Monte Carlo, p: p value for comparing between the studied groups, *Statistically significant at *p* ≤ 0.05.

Tooth brushing was reported by 82.5% of children with ECC and 82.5% of caries-free children (*p* = 0.953). Brushing teeth before bedtime was done by 57.6% of children with ECC and 75.8% of caries-free children (*p* = 0.117). Children use fluoridated toothpaste in 62.5% of cases and 57.5% in the controls group (*p* = 0.648). Parental supervision during tooth brushing was reported by 67.5% of children with ECC and 62.5% of caries-free children (*p* = 0.639).

### Clinical examination of the children and salivary cortisol levels

Table 4 shows the caries experience of children with ECC. The dmft index range was 1.0–20.0, with a mean ± SD of 6.83 ± 4.56. Table 4 also shows the Silness-Löe Plaque Index of children with ECC and caries-free children. The mean Plaque Index in children with ECC was significantly higher (1.28 ± 0.51) than that of caries-free children (0.34 ± 0.37) (*p* < 0.001). The mean salivary cortisol level in children with ECC (0.67 ± 0.23 µg/dl) is significantly higher than that of caries-free children (0.50 ± 0.22 µg/dl) (*p* = 0.003).

**Table 4:** Comparison between the two studied groups according to clinical examination of the child and salivary cortisol level (Medical Research Institute, 2023).

Clinical examination	Cases (*n* = 40)	Control (*n* = 40)	U	*p*-value
dmft index				
Min. – Max	1.0–20.0	0.0–0.0	0.001	< 0.001^*^
Mean ± SD	6.83 ± 4.56	0.0 ± 0.0		
Median (IQR)	6.0 (3.0–10.0)	0.0 ( – )		
Silness and Loe plaque index				
Min. – Max.	0.11–2.0	0.0–1.75	123.50	< 0.001^*^
Mean ± SD	1.28 ± 0.51	0.34 ± 0.37		
Median (IQR)	1.33 (1.0–1.75)	0.25 (0.04–0.52)		
Salivary cortisol level µg/dl				
Min. – Max.	0.16–0.99	0.26–0.90	487.0	0.003^*^
Mean ± SD.	0.67 ± 0.23	0.50 ± 0.22		
Median (IQR)	0.73 (0.60–0.81)	0.41 (0.33–0.65)		

U: Mann Whitney test, p: p value for comparing between the studied groups, *Statistically significant at *p* ≤ 0.05, SD: Standard deviation, IQR: Interquartile range.

### Receiver operating characteristic curve (ROC curve) for salivary cortisol level to predict cases

**Fig. 1:**
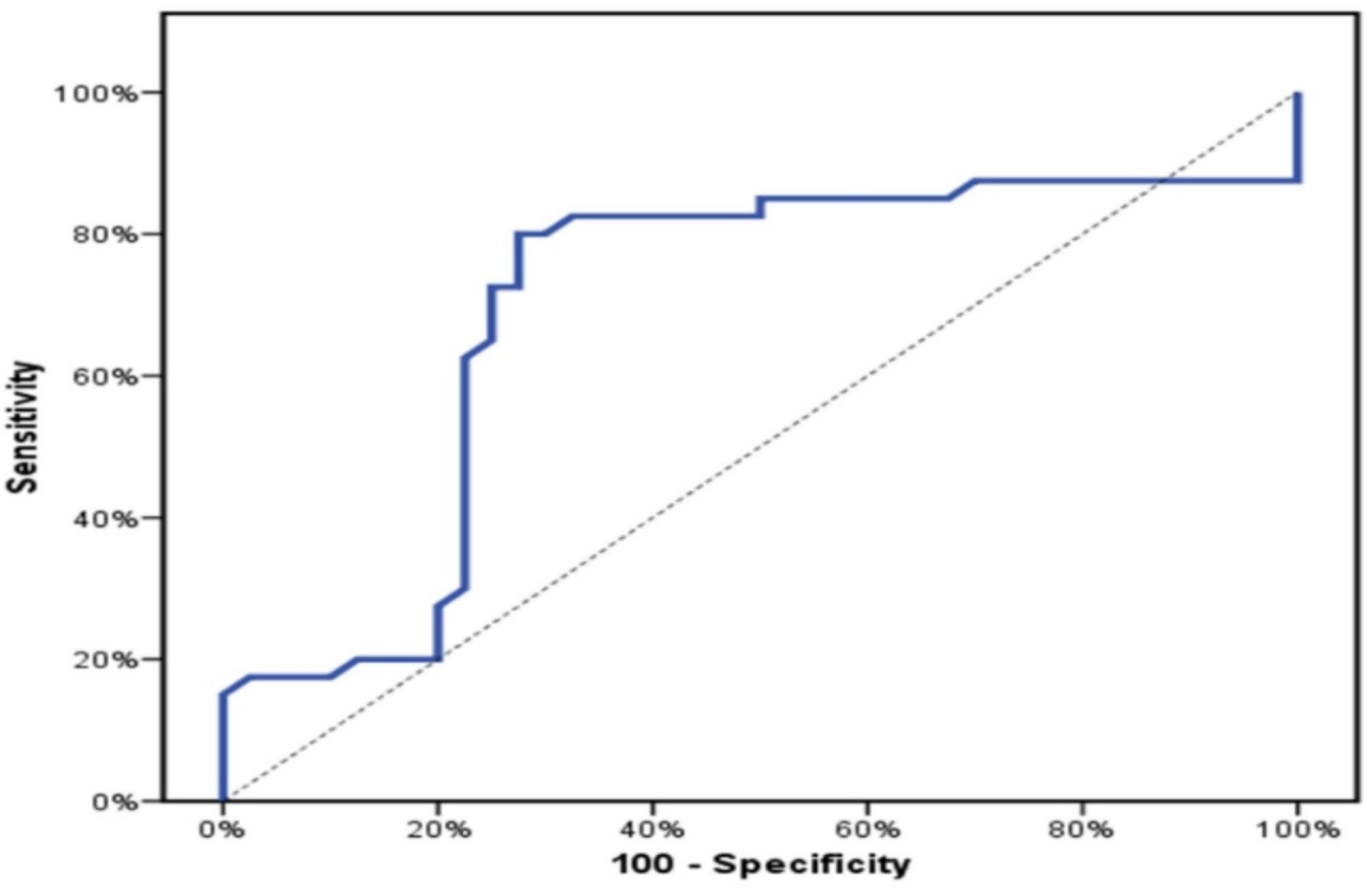
ROC curve for salivary cortisol level to predict cases.

The ROC curve was plotted to define the cut-off point for salivary cortisol level (Fig. 1). The Area Under the Curve was large enough (AUC) = 0.696; 95% CI = 0.571–0.820, *p* = 0.003) that salivary cortisol level can be considered a predictor of early childhood caries with a sensitivity of 80% and a specificity of 72.5%. The optimal cut-off point was found to be > 0.57 µg/dl (Table 5).

**Table 5:** Agreement (sensitivity, specificity) for salivary cortisol level to predict cases (Medical Research Institute, 2023).

	AUC	*P*-value	95% CI		Cut off	Sensitivity	Specificity	PPV	NPV
			L.L	U.L					
Salivary cortisol level	0.696^*^	0.003^*^	0.571	0.820	> 0.57	80.0	72.5	74.4	78.4

AUC: Area under a Curve, P value: Probability value, CI: Confidence Intervals, *Statistically significant at *p* ≤ 0.05, PPV: Positive predictive value, NPV: Negative predictive value.

80% of children with ECC had a salivary cortisol level > 0.57 µg/dl, which was more than double the percentage of caries-free children in the control group (27.5%) that had a salivary cortisol level > 0.57 µg/dl. The difference was statistically significant (*p* < 0.001). Concerning the risk of caries, children with a salivary cortisol level > 0.57 µg/dl revealed a significant risk for caries (Odds Ratio (OR) = 10.545; 95% confidence interval (CI): 3.73–29.84) compared to those with salivary cortisol ≤ 0.57 µg/dl (Table 6).

**Table 6:** Comparison between the two studied groups according to salivary cortisol cut off level (Medical Research Institute, 2023).

Salivary cortisol level	Cases (*n* = 40)		Control^®^ (*n* = 40)		OR	95%CI	*p*-value
	No.	%	No.	%			
≤ 0.57 µg/dl (-ve) ^®^	8	20.0	29	72.5			
> 0.57 µg/dl (+ ve)	32	80.0	11	27.5	10.545	3.73–29.840	< 0.001^*^

*OR* Odds ratio, *CI* Confidence interval, *LL* Lower limit, *UL* Upper Limit, ^®^: Reference group.

### Estimation of the association between caries in children and environmental stressors

Table 7 shows the results of the logistic regression analysis investigating the association between ECC and study variables: maternal education, paternal education, father smoking, having learning disabilities, total Epworth score, sleeping with a bottle, Silness-Löe Plaque Index, and salivary cortisol level > 0.57 µg/dl.

**Table 7:** Univariate and multivariate logistic regression analysis for the parameters affecting dental caries (Medical Research Institute, 2023).

	Univariate		Multivariate^#^	
	*p*-value	OR (95% CI)	*p*-value	OR (95% CI)
Mother working (non-working)	0.258	0.595 (0.242–1.462)		
Paternal education	0.644	1.238 (0.500–3.066)		
Father smoking	0.045^*^	2.513 (1.019–6.198)	0.030^*^	5.791 (1.183–28.336)
Having learning disabilities	0.038^*^	3.370 (1.070–10.613)	0.334	2.378 (0.410-13.782)
Total Epworth score	0.009^*^	1.138 (1.033–1.255)	0.528	1.056 (0.891–1.253)
Sleeping with bottle /food in the mouth	0.044^*^	4.111 (1.037–16.295)	0.027^*^	8.481 (1.272–56.554)
Silness-Löe Plaque Index	< 0.001^*^	8.671 (3.391–22.169)	0.001^*^	6.681 (2.239–19.931)
Salivary cortisol level (> 0.57 µg/dl)	< 0.001^*^	10.545 (3.73–29.840)	0.003^*^	9.649 (2.199–42.343)

*OR* Odds ratio, *CI* Confidence interval, *LL* Lower limit, *UL* Upper Limit, ^#^All variables with *p* < 0.05 was included in the multivariate, *Statistically significant at *p* ≤ 0.05.

In univariate regression, a significant association was observed with father smoking, having learning disabilities, total Epworth score, sleeping with a bottle or food in the mouth, Silness-Löe Plaque Index, and salivary cortisol level (> 0.57 µg/dl). Silness-Löe Plaque Index, and salivary cortisol level (> 0.57 µg/dl) were associated with higher odds of ECC (OR = 8.671 and 10.545).

All significant variables (*p* < 0.05) in the univariate analysis (father smoking, having learning disabilities, total Epworth score, sleeping with a bottle/food in the mouth, Silness-Löe Plaque Index, and elevated salivary cortisol) were included in a multivariate logistic regression model. This model revealed that only four factors were significantly associated with an increased risk of early childhood caries (ECC). The strongest predictor of ECC was elevated salivary cortisol levels (> 0.57 µg/dl) with an adjusted odds ratio (AOR) of 9.649 (*p* = 0.003). Other significant factors were father smoking (AOR = 5.791, *p* = 0.030), sleeping with a bottle or food in the mouth (AOR = 8.481, *p* = 0.027), and poor oral hygiene evaluated through the Silness-Löe Plaque Index (AOR = 6.681, *p* = 0.001).

## Discussion

Early childhood caries is a multifactorial disease and has numerous biological, psychological, behavioral, and socio-environmental risk factors^29^. Salivary cortisol has been used as a biomarker to explore the role of stress in children with ECC. Therefore, the goal of this study was to assess the salivary cortisol level as a stress biomarker in relation to environmental risk factors for ECC.

The present study revealed that the mean salivary cortisol level in children with ECC (0.67 ± 0.23 µg/dl) is significantly higher than that of caries-free children (0.50 ± 0.22 µg/dl) (*p* = 0.003). There is a significant association between free salivary cortisol and ECC at both univariate and multivariate logistic regression analysis. These results indicate that salivary cortisol is an independent stress biomarker in relation to early childhood caries. This is in accordance with another case-control study that stated that high salivary cortisol level was an independent factor associated with early childhood caries, with OR values of 3.05 (95% CI: 1.84–5.06) and 1.59 (95% CI: 1.09–2.58)^30^. There are also several studies that stated that salivary cortisol can be used as a tool to measure child’s stress in relation to ECC^12,20,31,32^.

According to the findings of our study, the statistically significant difference in parental education levels between cases and controls suggests that lower socioeconomic status, often indicated by lower education, may be a risk factor for childhood dental caries. This is similar to an Egyptian cross-sectional study that concluded that low parental education may be a risk factor for dental caries^19^. A Romanian study stated that the low level of education of parents was related to the high risk of caries among school children. The study concluded that parental education is reflected in the dental knowledge, behavior, and general care of the child and consequently contributes to the caries risk^33^.

Parents with lower education levels may have limited knowledge about oral health, leading to poor oral hygiene practices and dietary habits among their children. Additionally, socioeconomic challenges and increased stress experienced by these parents can indirectly impact their children’s oral health by elevating cortisol levels and compromising the oral defenses^15,34^.

Our data showed a statistically significant association between paternal smoking and a higher risk of dental caries in children. Children of smoking fathers were 2.51 times more likely to develop dental caries compared to children of non-smoking fathers (*p* = 0.046, 95% CI = 1.019–6.198). That is in accordance with other studies suggesting paternal smoking as a risk factor for ECC. Exposure to secondhand smoke can negatively impact a child’s oral health. While the exact mechanisms aren’t fully understood, it’s believed that secondhand smoke can reduce salivary flow, which has a vital role in washing away food particles and bacteria, increasing harmful bacteria, or impairing the immune system in the affected child^35–37^. Besides, exposure to secondhand smoke can lead to increased stress and elevated cortisol levels in children^38,39^.

Children with dental caries were more likely to have learning disabilities (*p* = 0.032) as shown in the present study. This was supported by a cross-sectional study that estimated the prevalence of dental caries in children with disabilities to be high^40^. On the other hand, a meta-analysis by Robertson et al. (2019) did not find a significant difference in caries levels between children with and without learning disabilities^41^. Some disabilities may make it challenging for children to understand the importance of oral hygiene or follow a consistent routine. Besides, children with certain disabilities might be sensitive to the taste of toothpaste, the feeling of a toothbrush, or the sound of dental equipment, making brushing a struggle. In addition, certain disabilities may be linked to weakened immune systems, making children more susceptible to oral infections that contribute to ECC^40^.

Children with learning disabilities often experience increased stress and elevated cortisol levels due to academic challenges, social difficulties, and the pressure to keep up with their peers. Chronic stress can negatively impact oral health by suppressing the immune system, altering salivary composition, and hindering the body’s natural repair processes. These factors can create an environment conducive to the growth of cariogenic bacteria, leading to increased susceptibility to dental caries^12,42^.

The present study revealed a significant difference in the quality of sleep between cases and controls, as measured by the Epworth Sleepiness Scale for Children and Adolescents (ESS-CHAD). To our knowledge, this is the first study to investigate the relationship between the ESS-CHAD and dental caries in children. A study stated that the risk of early childhood caries could correlate to sleep disturbances^43^, another study suggested that sleep problems among young children were a behavioral risk factor for night-time bottle use and early childhood caries^44^. In a Japanese population based cohort study, the Odds Ratios for children with short or irregular sleep duration compared with those with sleep duration of ≥ 11 h were 1.30, 1.16, 1.11, and 1.35 for sleep duration of ≤ 8, 9, 10 h, and irregular sleep duration, respectively, concluding that late bedtime and short sleep duration were both consistently associated with increased risk of caries in deciduous teeth^45^. Insufficient sleep can lead to elevated levels of stress hormones like cortisol which subsequently increase the risk of dental caries by promoting the growth of harmful bacteria, reducing saliva protective effects, and hindering the remineralization of tooth enamel^46,47^.

In our study, sleeping with bottle or food in the mouth was reported by 25% of children with ECC compared to only 7.5% of caries free children, the difference was statistically significant (*p* = 0.034). Children sleeping with bottle or food in their mouth had 4.11 times more risk to develop caries than those children did not have this habit (*p* = 0.044, 95% CI = 1.037–16.295). Sleeping with a bottle or food in the mouth significantly increases ECC because sugary liquids pool around teeth for extended periods, reducing saliva flow (which naturally cleans teeth) and allowing cavity-causing bacteria to thrive^48,49^.

The current study demonstrated that the plaque index is a risk factor for the development of caries (OR = 52.20, 95% CI = 10.63-256.34). This is in agreement with the results of a study conducted by Caruso et al. (2018), that showed that plaque build-up was a risk factor associated with caries presence, with an OR of 3.18 (95% CI: 1.13–8.91) for low plaque levels and an OR of 4.6 (95% CI: 1.48–14.20) for higher plaque levels^30^.

The relationship between stress and oral health is bidirectional. Stress can negatively impact oral health by elevating cortisol levels, which can suppress the immune system, alter salivary composition, and hinder the body’s natural repair processes. This can create an environment conducive to the growth of cariogenic bacteria, leading to increased susceptibility to dental caries and periodontal disease. Conversely, oral health problems, such as dental pain or periodontal disease, can exacerbate stress and anxiety, creating a vicious cycle^32^. Coxon et al. (2019) investigated the relationship between dental anxiety and oral health in children aged 5 and 8 years. The study found a strong association between dental anxiety and poorer oral health outcomes, including increased decay experience, active decay, and the need for dental restorations^50^. Although some studies have explored the relationship between dental caries, stress, and cortisol levels, further research is needed to solidify this connection. More studies are required to measure cortisol levels early before the development of dental caries and to monitor these levels throughout the disease progression and treatment process. This would provide a clearer understanding of the causal relationship between stress, cortisol, and the risk of dental caries in children.

## Limitations and strengths of the study

The present study has some limitations. First, in case-control studies, the possibility of recall bias can’t be avoided completely since the majority of exposure data was derived from self-reported history. The study’s design also made it more difficult to account for all possible confounders and assign causal links to the results that were discovered. Second, the possible impact of genetic heterogeneity on the relationship between risk factors and dental caries could not be ascertained due to the absence of genetic information for the studied participants. Third, the small sample size may make it challenging to ascertain whether a given result is a trustworthy finding. Further research with larger sample sizes is needed to confirm our results. Additionally, long-term follow-up studies are necessary to evaluate the long-term impact of early childhood stress on oral health outcomes. On the other hand, this study has many points of strength. First, the present research studied the environmental stressors of ECC, which were seldom studied in Egypt. In addition, it measured the salivary cortisol level, which is considered an objective indicator of stress that could affect the oral health of children. Besides, it calculated the cut-off level of salivary cortisol; above it, the risk for ECC increased significantly. We used various validated tools to obtain scientifically sound results and to overcome the study limitations.

## Conclusion & recommendations

Based on the results of the current case-control study, we can conclude that the salivary cortisol level as a stress biomarker may be a significant risk factor associated with the development of early childhood caries. The significant association between the salivary cortisol level and ECC at both univariate and multivariate logistic regression analysis indicates that salivary cortisol is an independent stress biomarker in relation to ECC.

The mean pediatric Epworth sleepiness score in children with ECC was significantly higher than that of caries-free children, shedding light on the role of sleep disturbance in enhancing ECC. Parental education is reflected in the dental knowledge, behavior, and general care of the child and consequently contributes to caries risk. Paternal smoking, children with learning disabilities, children sleeping with a bottle or food in their mouth, or a high dental plaque index increase the risk of ECC.

In light of the results of the present study, it is recommended that pediatric dentists and public health specialists should collaborate to establish education sessions for family members and caregivers in the kindergartens, schools, and clubs to raise their knowledge and awareness about environmental stress factors and their relation to ECC and how to reduce them. They should also help teachers recognize psychological health problems among the children whom they teach to enable timely detection of stress factors at school and the initiation of the necessary protective measures.

## Electronic supplementary material

Below is the link to the electronic supplementary material.

Supplementary Material 1

Supplementary Material 2

Supplementary Material 3

## Data Availability

The datasets used and/or analyzed during the current study are available from the corresponding author on reasonable request.

## Abbreviations

AOR: Adjusted odds ratio
AUC: Area under the curve
CI: Confidence interval
dmft: Decayed, missing, and filled teeth
ECC: Early childhood caries
ESS-CHAD: Epworth sleepiness scale for children and adolescents
MS: Mutans streptococci
OR: Odds ratio
P: p value
ROC: Receiver operating characteristic curve
SD: Standard deviation
U: Mann Whitney test
WHO: World Health Organization

## References

[1] Benjamin, R. M. Oral health: the silent epidemic. *Public Health Rep.* **125** (2), 158–159. 10.1177/003335491012500202 (2010). 20297740 10.1177/003335491012500202 PMC2821841

[2] Niskanen, M. C., Mattila, P. T., Niinimaa, A. O., Vehkalahti, M. M. & Knuuttila, M. L. Behavioural and socioeconomic factors associated with the simultaneous occurrence of periodontal disease and dental caries. *Acta Odontol. Scand.* **78** (3), 196–202. 10.1080/00016357.2019.1679389 (2020). 31686553 10.1080/00016357.2019.1679389

[3] Pitts, N. B. et al. Dental caries. *Nat. Rev. Dis. Primers*. **3** (1), 1–6. 10.1038/nrdp.2017.30 (2017). 10.1038/nrdp.2017.30 28540937

[4] Kazeminia, M. et al. Dental caries in primary and permanent teeth in children’s worldwide, 1995 to 2019: a systematic review and meta-analysis. *Head Face Med.* **16** (1), 22. 10.1186/s13005-020-00237-z (2020). 33023617 10.1186/s13005-020-00237-z PMC7541284

[5] Wong, H. M. Childhood caries management. *Int. J. Environ. Res. Public Health*. **19** (14), 8527. 10.3390/ijerph19148527 (2022). 35886380 10.3390/ijerph19148527 PMC9321968

[6] Baghdadi, Z. D. Early childhood caries and Indigenous children in Canada: prevalence, risk factors, and prevention strategies. *J. Int. Oral Health*. **8** (7), 830–837. 10.2047/jioh-08-07-17 (2016).

[7] Anil, S. & Anand, P. S. Early childhood caries: prevalence, risk factors, and prevention. *Front. Pead.* **5**, 157. 10.3389/fped.2017.00157 (2017). 10.3389/fped.2017.00157 PMC5514393 28770188

[8] Carvalho, T. S., Lussi, A., Schlueter, N. & Baumann, T. Differences in susceptibility of deciduous and permanent teeth to erosion exist, albeit depending on protocol design and method of assessment. *Sci. Rep.* **12** (1), 4153. 10.1038/s41598-022-08116- (2022). 35264778 10.1038/s41598-022-08116-0 PMC8907165

[9] Folayan, M. O. et al. Association between environmental health, ecosystem vitality, and early childhood caries. *Front. Pead.* **8**, 196. 10.3389/fped.2020.00196 (2020). 10.3389/fped.2020.00196 PMC7248316 32509710

[10] Nadeeshani, H., Kudagammana, S. T., Herath, C., Jayasinghe, R. & Liyanage, R. Early childhood caries and nutritional status of children: A review. *FoodNutr. Bull.* **44** (4), 249–264. 10.1177/03795721231209358 (2023). 10.1177/03795721231209358 38095292

[11] Çolak, H., Dülgergil, Ç. T., Dalli, M. & Hamidi, M. M. Early childhood caries update: A review of causes, diagnoses, and treatments. *J. Nat. Sci. Biology Med.* **4** (1), 29–38. 10.4103/0976-9668.107257 (2013). 10.4103/0976-9668.107257 PMC3633299 23633832

[12] Padmanabhan, V. et al. Association between salivary cortisol levels, dental anxiety, and dental caries in children: A cross-sectional study. *Dentistry J.* **11** (9), 205. 10.3390/dj11090205 (2023). 10.3390/dj11090205 PMC10528522 37754325

[13] Mohiti, A., Ardakani, M. E. & Amooei, M. The effect of systemic corticosteroid use on the pH and viscosity of saliva. *Shiraz E Med. J.* **22** (4). 10.5812/semj.101710 (2021).

[14] Magacz, M., Kędziora, K., Sapa, J. & Krzyściak, W. The significance of lactoperoxidase system in oral health: application and efficacy in oral hygiene products. *Int. J. Mol. Sci.* **20** (6), 1443. 10.3390/ijms20061443 (2019). 30901933 10.3390/ijms20061443 PMC6472183

[15] Tarullo, A. R., Tuladhar, C. T., Kao, K., Drury, E. B. & Meyer, J. Cortisol and socioeconomic status in early childhood: A multidimensional assessment. *Dev. Psychopathol.* **32** (5), 1876–1887. 10.1017/S0954579420001315 (2020). 33427182 10.1017/S0954579420001315 PMC7938639

[16] Agarwal, S., Chandak, M., Reche, A. & Singh, P. V. The prevalence of dental fear and its relationship to dental caries and gingival diseases among school children in Wardha. *Cureus* **15** (10). 10.7759/cureus.46360 (2023). 10.7759/cureus.46360 PMC10619468 37920631

[17] Hamzany, Y. et al. Is human saliva an indicator of the adverse health effects of using mobile phones? *Antioxid. Redox. Signal.* **18** (6), 622–627. 10.1089/ars.2012.4751 (2013). 22894683 10.1089/ars.2012.4751

[18] World Health Organization. Egypt releases results of epidemiological study on oral health status. WHO. (2014). http://www.emro.who.int/egy/egypt-events/results-of-epidemiological-study-on-oral-health-status-released.html

[19] Abbass, M. M. et al. The prevalence of dental caries among Egyptian children and adolescences and its association with age, socioeconomic status, dietary habits and other risk factors. A cross-sectional study. *F1000Research* 8. 10.12688/f1000research.17047.1 (2019). 10.12688/f1000research.17047.1 PMC6396843 30854195

[20] Rai, K., Hegde, A. & Shetty, S. Estimation of salivary cortisol in children with rampant caries. *J. Clin. Pediatr. Dentistry*. **34** (3), 249–252. 10.17796/jcpd.34.3.l858480k80031jn2 (2010). 10.17796/jcpd.34.3.l858480k80031jn2 20578663

[21] Gao, X. et al. Validity of caries risk assessment programmes in preschool children. *J. Dent.* **41** (9), 787–795. 10.1016/j.jdent.2013.06.005 (2013). 23791698 10.1016/j.jdent.2013.06.005

[22] Laksmiastuti, S. R., Budiardjo, S. B. & Sutadi, H. Validated questionnaire of maternal attitude and knowledge for predicting caries risk in children: epidemiological study in North Jakarta, Indonesia. *J. Int. Soc. Prev. Community Dentistry*. **7** (Suppl 1), S42–S47. 10.4103/jispcd.JISPCD_148_17 (2017). 10.4103/jispcd.JISPCD_148_17 PMC5502551 28713767

[23] Fontana, M. et al. Predicting caries in medical settings: risk factors in diverse infant groups. *J. Dent. Res.* **98** (1), 68–76. 10.1177/0022034518799080 (2019). 30205016 10.1177/0022034518799080 PMC6304713

[24] Janssen, K. C., Phillipson, S., O’Connor, J. & Johns, M. W. Validation of the Epworth sleepiness scale for children and adolescents using Rasch analysis. *Sleep. Med.* **33**, 30–35. 10.1016/j.sleep.2017.01.014 (2017). 28449902 10.1016/j.sleep.2017.01.014

[25] World Health Organization. Mean number of decayed, missing, and filled permanent teeth (mean DMFT) among the 12-year-old age group. *WHO* (2016). https://www.who.int/data/gho/indicator-metadata-registry/imr-details/3812

[26] Silness, J. & Löe, H. Periodontal disease in pregnancy II. Correlation between oral hygiene and periodontal condition. *Acta Odontol. Scand.* **22** (1), 121–135. 10.3109/00016356408993968 (1964). 14158464 10.3109/00016356408993968

[27] Kelly, S. J., Young, R., Sweeting, H., Fischer, J. E. & West, P. Levels and confounders of morning cortisol collected from adolescents in a naturalistic (school) setting. *Psychoneuroendocrinology* **33** (9), 1257–1268. 10.1016/j.psyneuen.2008.06.010 (2008). 18691824 10.1016/j.psyneuen.2008.06.010 PMC2571963

[28] Kalman, B. A. & Grahn, R. E. Measuring salivary cortisol in the behavioral neuroscience laboratory. *J. Undergrad. Neurosci. Educ.* **2** (2), A41 (2004). 23493518 PMC3592595

[29] Sridevi, T., Pranoti, S., Anand, S., Umesh, W. & Sachin, G. Factors associated with early childhood caries among 3 to 6 year old children in India: a case control study. *J. Neonatal-Perinatal Med.* **11** (1), 45–50. 10.3233/NPM-181723 (2018). 29689741 10.3233/NPM-181723

[30] Caruso, S., Gatto, R., Cinque, B., Cifone, M. G. & Mattei, A. Association between salivary cortisol level and caries in early childhood. *Eur. J. Paediatr. Dent.* **19**, 10–15. 10.23804/ejpd.2018.19.01.02 (2018). 29569447 10.23804/ejpd.2018.19.01.02

[31] Rani, S. P., Amrutha, C., Anantharaj, A., Praveen, P. & Sudhir, R. Salivary cortisol: A biomarker for stress indicator in children. *CODS-Journal Dentistry*. **12** (1), 7–10. 10.5005/jp-journals-10063-0064 (2021).

[32] Pani, S. C., Abuthuraya, D., AlShammery, H. M., AlShammery, D. & AlShehri, H. Salivary cortisol as a biomarker to explore the role of maternal stress in early childhood caries. *Int. J. Dent.* (2013). 10.1155/2013/565102 10.1155/2013/565102 PMC3679687 23781246

[33] Dumitrescu, R. et al. The impact of parental education on schoolchildren’s oral health—a multicenter cross-sectional study in Romania. *Int. J. Environ. Res. Public Health*. **19** (17), 11102. 10.3390/ijerph191711102 (2022). 36078817 10.3390/ijerph191711102 PMC9518154

[34] Almajed, O. S., Aljouie, A. A., Alharbi, M. S. & Alsulaimi, L. M. The impact of socioeconomic factors on pediatric oral health: A review. *Cureus* **16** (2), e53567. 10.7759/cureus.53567 (2024). 10.7759/cureus.53567 PMC10914081 38445162

[35] Shenkin, J. D., Broffitt, B., Levy, S. M. & Warren, J. J. The association between environmental tobacco smoke and primary tooth caries. *J. Public Health Dent.* **64** (3), 184–186. 10.1111/j.1752-7325.2004.tb02750.x (2004). 15341143 10.1111/j.1752-7325.2004.tb02750.x

[36] Mosharrafian, S., Lohoni, S. & Mokhtari, S. Association between dental caries and passive smoking and its related factors in children aged 3–9 years old. *Int. J. Clin. Pediatr. Dentistry*. **13** (6), 600. 10.5005/jp-journals-10005-1831 (2020). 10.5005/jp-journals-10005-1831 PMC8060945 33976482

[37] Hanioka, T., Nakamura, E., Ojima, M., Tanaka, K. & Aoyama, H. Dental caries in 3-year‐old children and smoking status of parents. *Paediatr. Perinat. Epidemiol.* **22** (6), 546–550 (2008). 19000292 10.1111/j.1365-3016.2008.00950.x

[38] Arafa, A. Household smoking impact on the oral health of 5- to 7-years-old children. *BMC Oral Health*. **23** (1), 1028. 10.1186/s12903-023-03715-3 (2023). 38114982 10.1186/s12903-023-03715-3 PMC10731723

[39] Misrabi, G., Karkoutly, M. & Bshara, N. The effect of secondhand smoke exposure on dental caries and gingival health among schoolchildren in Damascus, Syria: a cross-sectional study. *BMC Oral Health*. **23** (1), 745. 10.1186/s12903-023-03486-x (2023). 37821880 10.1186/s12903-023-03486-x PMC10568839

[40] Uwayezu, D., Gatarayiha, A. & Nzayirambaho, M. Prevalence of dental caries and associated risk factors in children living with disabilities in Rwanda: a cross-sectional study. *Pan Afr. Med. J.* **36** (1), 193. 10.11604/pamj.2020.36.193.24166 (2020). 32952837 10.11604/pamj.2020.36.193.24166 PMC7467614

[41] Robertson, M. D. et al. Dental caries experience, care index and restorative index in children with learning disabilities and children without learning disabilities; a systematic review and meta-analysis. *BMC Oral Health*. **19**, 1–6. 10.1186/s12903-019-0795-4 (2019). 31307444 10.1186/s12903-019-0795-4 PMC6632188

[42] Burenkova, O. V., Naumova, O. Y. & Grigorenko, E. L. Stress in the onset and aggravation of learning disabilities. *Dev. Rev.* **61**, 100968. 10.1016/j.dr.2021.100968 (2021). 34219858 10.1016/j.dr.2021.100968 PMC8244356

[43] Arroyo Buenestado, A. & Ribas-Pérez, D. Early childhood caries and sleep disorders. *J. Clin. Med.* **12** (4), 1378. 10.3390/jcm12041378 (2023). 36835914 10.3390/jcm12041378 PMC9967236

[44] Shantinath, S. D., Breiger, D., Williams, B. J. & Hasazi, J. E. The relationship of sleep problems and sleep-associated feeding to nursing caries. *Pediatr. Dent.* **18**, 375–378 (1996). 8897529

[45] Chen, H., Tanaka, S., Arai, K., Yoshida, S. & Kawakami, K. Insufficient sleep and incidence of dental caries in deciduous teeth among children in Japan: a population-based cohort study. *J. Pediatr.* **198**, 279–286. 10.1016/j.jpeds.2018.03.033 (2018). 29709344 10.1016/j.jpeds.2018.03.033

[46] Moro, J., Santos, P., Giacomin, A., Cardoso, M. & Bolan, M. Association between trouble sleeping and oral conditions among schoolchildren. *Rev. Paulista Pediatria*. **39**, e2019342. 10.1590/1984-0462/2021/39/2019342 (2020). 10.1590/1984-0462/2021/39/2019342 PMC7518721 32996996

[47] Nollet, M., Wisden, W. & Franks, N. P. Sleep deprivation and stress: a reciprocal relationship. *Interface Focus*. **10** (3), 20190092. 10.1098/rsfs.2019.0092 (2020). 32382403 10.1098/rsfs.2019.0092 PMC7202382

[48] Barjatya, K., Nayak, U. A. & Vatsal, A. Association between early childhood caries and feeding practices among 3–5-year-old children of Indore, India. *J. Indian Soc. Pedod. Prev. Dentistry*. **38** (2), 98–103. 10.4103/JISPPD.JISPPD_60_20 (2020). 10.4103/JISPPD.JISPPD_60_20 32611852

[49] Khodke, S., Naik, S. & Agarwal, N. Infant dietary pattern and its association with early childhood caries in preschool children: A Cross-sectional study. *Int. J. Clin. Pediatr. Dentistry*. **16** (3), 421. 10.5005/jp-journals-10005-2356 (2023). 10.5005/jp-journals-10005-2356 PMC10367304 37496948

[50] Coxon, J. D., Hosey, M. T. & Newton, J. T. The impact of dental anxiety on the oral health of children aged 5 and 8 years: a regression analysis of the child dental health survey 2013. *Br. Dent. J.* **227** (9), 818–822. 10.1038/s41415-019-0853-y (2019). 31705101 10.1038/s41415-019-0853-y

