# Supplementary material for: Association between environmental stress factors, salivary cortisol level and dental caries in Egyptian preschool children: a case-control study
Source: Sci Rep. 2025 Apr 1;15:11063. doi: 10.1038/s41598-025-94327-0 (PMC11961556; doi:10.1038/s41598-025-94327-0)
Supplement: Supplementary file 1 — Supplementary Material 1 [file 41598_2025_94327_MOESM1_ESM.docx]

# Supplementary File I - English Translation

## Data Collection Questionnaire

## Alexandria University

## High Institute of Public Health

## Questionnaire Number:

## Date:

## Belongs to:

(1) Case Group (0) Control Group

## Section One: Personal Information

## 1. Name:

## 2. Age:

## 3. Gender:

(1) Male (2) Female

## 4. Current Address:

1) Montazah District, 2) Sharq District, 3) Wasat District, 4) Gharb District, 5) Gomrok District, 6) Amreya District, 7) Dekheila District, 8) Borg El Arab City

## 5. Birth Order Among Siblings:

1) First, 2) Second, 3) Third, 4) More than Third

## 6. Mother's Educational Level:

(01) Illiterate (02) Reads and Writes (03) Completed Primary Education (04) Completed Preparatory Education (05) Completed Secondary/Vocational Education (06) Completed Post-secondary Education (07) Completed University Education (08) Postgraduate

## 7. Mother's Occupation:

(01) Housewife (02) Office Worker (03) Unskilled Worker (04) Skilled Worker (05) Agricultural Work (06) Industrial Work (07) Commercial Work (08) Medical Field (09) Specialized Profession (10) Other (please specify)

## 8. Father's Educational Level:

(01) Illiterate (02) Reads and Writes (03) Completed Primary Education (04) Completed Preparatory Education (05) Completed Secondary/Vocational Education (06) Completed Post-secondary Education (07) Completed University Education (08) Postgraduate

## 9. Father's Occupation:

(01) Unemployed (02) Office Worker (03) Unskilled Worker (04) Skilled Worker (05) Agricultural Work (06) Industrial Work (07) Commercial Work (08) Medical Field (09) Specialized Profession (10) Military/Police (11) Transportation Work (12) Self-employed (13) Other (please specify)

## Section Two: Questions on Smoking

## 10. Mother's Smoking Habits:

1) Number of cigarettes/packs per day, 2) Number of years of smoking, (00) Mother does not smoke

## 11. Father's Smoking Habits:

1) Number of cigarettes/packs per day, 2) Number of years of smoking, (00) Father does not smoke

## 12. Presence of Smokers in the House:

(0) No (01) Mother (02) Father (03) Both Mother and Father (04) Others

## 13. Does the mother smoke shisha?

(01) No (02) Yes

## 14. Does the mother smoke other types (e.g., e-cigarettes)?

(01) No (02) Yes

## 15. Does the father or others smoke shisha?

(01) No (02) Yes

## 16. Does the father smoke other types (e.g., e-cigarettes)?

(01) No (02) Yes

## Section Three: Questions on Potential Psychological and Social Stress in the Child's Daily Environment

## Family Life Questions

## 17. Household Income:

(01) Sufficient and more (02) Sufficient only (03) Insufficient

## 18. Do any of the parents suffer from depression?

(01) No (02) Yes

## 19. Is there separation/divorce between the parents?

(01) No (02) Yes

## 20. Are there conflicts or violence between the parents?

(01) No (02) Yes

## 21. Does the mother face any of the following frequently at work?

1) Psychological or nervous stress, 2) Physically demanding work, 3) Long hours of standing, 4) Night shifts, 5) Other factors (please specify), (-1) Does not work

## 22. Is the child subjected to neglect or abuse?

(01) No (02) Yes

## 23. Is there a new sibling for the child?

(01) No (02) Yes

## 24. Has there been a death in the family?

(01) No (02) Yes

## 25. Are there fights or conflicts with siblings?

(01) No (02) Yes

## 26. Are there too many tasks for the child to complete?

(01) No (02) Yes

## Questions About the Child’s Kindergarten or School Environment

## 27. Discrimination by teachers:

(01) No (02) Yes

## 28. Bullying by other children at school:

(01) No (02) Yes

## 29. Losing toys:

(01) No (02) Yes

## 30. Difficulty making friends or experiencing racism:

(01) No (02) Yes

## 31. Learning difficulties:

(01) No (02) Yes

## 32. Transferring to another school:

(01) No (02) Yes

## 33. Poor grades:

(01) No (02) Yes

## 34. Too much homework:

(01) No (02) Yes

## Questions About the Child's Exposure to Electromagnetic Waves Inside or Outside the Home

## 35. Does the child or school reside near high voltage lines?

(1) No (2) Yes (-2) Unknown

## 36. Does the child live near a radio or TV broadcasting station?

(1) No (2) Yes (-2) Unknown

## 37. Does the child live near a mobile phone tower?

(1) No (2) Yes (-2) Unknown

## 38. Are there electronic devices or video games at home?

(01) No (02) Yes

## 39. At what age did your child first engage in activities on electronic devices?

Activities: Touching or swiping the screen, making a call, playing video games, watching TV programs, using apps.

## Daily exposure duration to any of the following electronic devices:

## 40. Television:

(1) Not available (2) Less than 2 hours (3) 2-5 hours (4) More than 5 hours

## 41. Mobile phone:

(1) Not available (2) Less than 10 minutes (3) 10-30 minutes (4) More than 30 minutes

## 42. Computer/video games:

(1) Not available (2) Less than 2 hours (3) 2-5 hours (4) More than 5 hours

## 43. How long was your child exposed to electronic devices yesterday?

(1) Not available (2) 15 minutes (3) 30 minutes (4) 60 minutes (5) 90 minutes (6) More than 90 minutes

## 44. What types of apps were downloaded for your child?

Educational (to enhance cognitive development), Entertainment (for fun, any cognitive benefit was secondary).

## 45. Does your child use more than one device at the same time?

(01) No (02) Sometimes (03) Often

## 46. Does your child eat while using devices?

(01) No (02) Yes

## Epworth Sleepiness Scale (Adapted for Children):

Situation - Chance of dozing or sleeping:

## 47. Sitting and reading:

## 0 ) would never fall asleep

## 1 ) slight chance of falling asleep

## 2 ) moderate chance of falling asleep

## 3 ) high chance of falling asleep

## 48. Sitting and Watching TV or video:

0) would never fall asleep

1) slight chance of falling asleep

2) moderate chance of falling asleep

3) high chance of falling asleep

**49. Sitting in a classroom at school during morning:**

0) would never fall asleep

1) slight chance of falling asleep

2 ) moderate chance of falling asleep

3 ) high chance of falling asleep

**50. Sitting in a car or bus for about half an hour:**

0) would never fall asleep

1) slight chance of falling asleep

2) moderate chance of falling asleep

3) high chance of falling asleep

**51. Lying down to rest or nap in the afternoon:**

0) would never fall asleep

1) slight chance of falling asleep

2 ) moderate chance of falling asleep

3 ) high chance of falling asleep

**52. Sitting and talking to someone:**

0) would never fall asleep

1) slight chance of falling asleep

2 ) moderate chance of falling asleep

3 ) high chance of falling asleep

**53. Sitting quietly by yourself after lunch:**

0) would never fall asleep

1 ) slight chance of falling asleep

2) moderate chance of falling asleep

3 ) high chance of falling asleep

**54. Sitting quietly by yourself after lunch:**

0) would never fall asleep

1) slight chance of falling asleep

2 ) moderate chance of falling asleep

3 ) high chance of falling asleep

**55. Total Score on the Epworth Sleepiness Scale:**

- **Dental History and Oral Health**

**56. Previous visit to a dental clinic:**

(01) No (02) Yes

**57. Feeding:**

(01) Breastfeeding (02) Formula feeding

**58. Does the child sleep with a bottle or food in their mouth?**

(01) No (02) Yes

**59. Does the child wake up to eat or drink juice or water?**

(01) No (02) Yes

**60. Does the child brush their teeth before bed?**

(01) No (02) Yes

**61. Brushing teeth with a toothbrush:**

(01) No (02) Once daily (03) Twice daily

**62. Using fluoride toothpaste:**

(01) No (02) Yes

**63. Parental supervision of tooth brushing:**

(01) No (02) Yes

- **Questions on the Child's Nutrition:**

**64. Does the child eat between meals?**

(01) No (02) Once daily (03) Twice daily (04) More often

**65. Does the child eat sweets?**

(1) No (2) Once weekly (3) 2-5 times weekly (4) Daily

**66. Does the child eat chips?**

(1) No (2) Once weekly (3) 2-5 times weekly (4) Daily

**67. Does the child eat fresh fruits and vegetables?**

(1) No (2) Once weekly (3) 2-5 times weekly (4) Daily

**68. Does the child drink soft drinks?**

(1) No (2) Once weekly (3) 2-5 times weekly (4) Daily

**69. Does the child consume dairy products or drink milk?**

(1) No (2) Once weekly (3) 2-5 times weekly (4) Daily

**70. Does the child eat fish?**

(1) No (2) Once weekly (3) 2-5 times weekly (4) Daily

- **Child's Examination Data**

**71. Dental caries (DMFT Index):**

**72. Oral hygiene (Silness and Loe Plaque Index):**

**73. Salivary cortisol level:**
