# Supplementary material for: Association between environmental stress factors, salivary cortisol level and dental caries in Egyptian preschool children: a case-control study
Source: Sci Rep. 2025 Apr 1;15:11063. doi: 10.1038/s41598-025-94327-0 (PMC11961556; doi:10.1038/s41598-025-94327-0)
Supplement: Supplementary file 2 — Supplementary Material 2 [file 41598_2025_94327_MOESM2_ESM.docx]

**Epworth Sleepiness Scale for Children and Adolescents**

**(ESS-CHAD)**

Your name:___________________________ Today’s date:______________

How old are you? ______ (years) Boy? ( ____ ) or Girl? ( ____ ) tick one space

Over the last month, how likely have you been to fall asleep while doing the things that are described below (activities)?

Even if you haven’t done some of those things over the last month, try to imagine how they would have affected you.

Use the following scale to choose one number that best describes what has been happening to you during each activity over the last month. Write that number in the box below.

0 = would never fall asleep

1 = slight chance of falling asleep

2 = moderate chance of falling asleep

3 = high chance of falling asleep

It is important that you answer each question as best you can

| Activities | Chance of falling asleep 0-3 |
| --- | --- |
| Sitting and reading |  |
| Sitting and watching TV or a video |  |
| Sitting in a classroom at school during morning |  |
| Sitting in a car or bus for about half an hour |  |
| Lying down to rest or nap in the afternoon |  |
| Sitting and talking to someone |  |
| Sitting quietly by yourself after lunch |  |
| Sitting and eating a meal |  |
| Total |  |
