# Supplementary material for: Association between environmental stress factors, salivary cortisol level and dental caries in Egyptian preschool children: a case-control study
Source: Sci Rep. 2025 Apr 1;15:11063. doi: 10.1038/s41598-025-94327-0 (PMC11961556; doi:10.1038/s41598-025-94327-0)
Supplement: Supplementary file 3 — Supplementary Material 3 [file 41598_2025_94327_MOESM3_ESM.docx]

**Table1: The Silness-Löe Plaque Index System**

| **Scores** | **Criteria** | |
| --- | --- | --- |
| 0 | No plaque | |
| 1 | A film of plaque adhering to the free gingival margin and adjacent area of the tooth. The plaque may be seen in situ only after application of disclosing solution or by using the probe on the tooth surface. | |
| 2 | Moderate accumulation of soft deposit s within the gingival pocket, or the tooth and gingival margin which can be seen with the naked eye. | |
| 3 | | Abundance of soft matter within the gingival pocket and/or on the tooth and gingival margin. |
